# The role of gut microbiota and plasma metabolites in osteoporosis: Insights from Mendelian randomization analysis

**DOI:** 10.1097/MD.0000000000044409

**Published:** 2025-09-12

**Authors:** WeiDong Zhu, ShuWei Ma, XuWen Zheng, JinNan Yin

**Affiliations:** a Emergency Department, Wujin People’s Hospital Affiliated with Jiangsu University and Wujin Clinical College of Xuzhou Medical University, Changzhou, Jiangsu, China; b Department of Spine Surgery, the Ninth Medical Center, Chinese PLA General Hospital, Beijing, China.

**Keywords:** gut microbiota, Mendelian randomization, meta-analysis, osteoporosis, plasma metabolites

## Abstract

Studies have implicated the gut microbiota as significant in bone condition, influencing osteoporosis through immune modulation, endocrine regulation, and metabolite production. Our study explores the plasma metabolites’ mediation effect between gut microbiomes and osteoporosis through Mendelian randomization (MR). Using publicly accessible GWAS data from 5959 individuals for gut microbiota and 8299 individuals for plasma metabolites, we employed MR analysis to explore their causal effects on osteoporosis. Osteoporosis outcome data were obtained from Pan-UKB, GERA, and FinnGen, covering 21,353 cases and 853,313 controls. Mediation effects of identified bacterial taxa on osteoporosis through plasma metabolites were computed using the product-of-coefficients approach. Our MR analysis identified several gut microbiomes and plasma metabolites potentially associated with osteoporosis. Notably, increased abundances of certain gut microbiomes like *Desulfobacterota* were linked with higher osteoporosis risk. Mediation analysis revealed that specific plasma metabolites like 5alpha-androstan-3alpha,17beta-diol monosulfate (3α-diol MS) levels significantly mediated the effects of these microbiomes on osteoporosis, explaining up to 9.15% of their effect. This study confirms the profound impact of gut microbiota on osteoporosis risk, mediated through specific plasma metabolites. These findings enhance our comprehension of the microbiota–bone health axis and could lead to new biomarkers or therapeutic targets for osteoporosis management, emphasizing the potential for modifying gut microbiota to mitigate disease risk.

## 1. Introduction

Osteoporosis is a significant public health concern that is defined by a reduction and deterioration of bone mass, leading to an increased susceptibility to fractures and fragility. It is primarily found in the elderly, with a significant increase in incidence observed in women following menostasis and in men around 65 years old.^[1]^ Clinically, osteoporosis frequently remains asymptomatic until a fracture occurs. Osteoporosis is frequently linked to fractures of the hip, vertebrae, and wrist. These fractures result in substantial morbidity, a reduced quality of life, and an increase in mortality.^[2]^ The societal impact of osteoporosis is profound, encompassing substantial healthcare costs, loss of productivity, and long-term disability.^[1]^ Osteoporosis is a multifactorial condition that is caused by a combination of genetic, hormonal, and environmental factors. It is characterized by an imbalance between osteoclasts’ bone resorption and osteoblasts’ bone generation at the cellular level. Genetic predisposition plays a critical role, influencing bone density and quality. Hormonal changes, particularly decreased estrogen levels in postmenopausal women, significantly accelerate bone loss. Additionally, nutritional deficiencies, sedentary lifestyle, and certain medications facilitate the onset and advancement of osteoporosis.^[3]^

The association between gastrointestinal microbiomes and osteoporosis has become a prominent area to be studied nowadays. Research have implicated that the gut microbiota as significant gut microbiota plays a crucial role in bone metabolic processes through various ways such as immune modulation, endocrine regulation, and metabolite production.^[4]^ The gut microbiota influences the balanced state between pro-inflammatory and anti-inflammatory cytokines, which in turn affects bone resorption and formation. For instance, dysbiosis in the gut bacteria may result in elevated levels of TNF-α and IL-6, promoting osteoclast activity and bone loss.^[5]^ Additionally, specific gastrointestinal microorganisms have been shown to generate short-chain fatty acids that possess a positive influence on bone health by stimulating the differentiation of osteoblasts and inhibiting osteoclast activity.^[6]^ While it is well established that gut microbiota influences the host through its metabolites, the specific metabolites impacting osteoporosis remain unclear. Gut-derived metabolites such as short-chain fatty acids, bile acids, and tryptophan metabolites are known to affect bone health, but their precise roles and mechanisms in osteoporosis are not fully understood.^[7]^

To address this gap in knowledge, we plan to perform a mediation Mendelian randomization (MR) analysis, which is a statistical technique used in epidemiology and genetics that employs genetic variants as instrumental variables to assess the causal effect of a modifiable exposure on an outcome. The core principle of MR is that genetic variations, randomly assigned at conception, can be utilized to infer causality between exposures and outcomes. This approach minimizes confounding factors and reverse causation, provided specific assumptions are met.^[8]^ We aim to determine whether specific plasma metabolites mediate the relationship between the gastrointestinal microbiomes and osteoporosis using MR. By examining the effects of genetic variations on both the gastrointestinal microbiota and plasma metabolites, we hope to gain a more comprehensive understanding of their roles in osteoporosis. This insight has the potential to revolutionize the management of osteoporosis by facilitating the generation of innovative diagnostic and treatment approaches.

## 2. Methods

### 2.1. Study design

**Figure 1.**
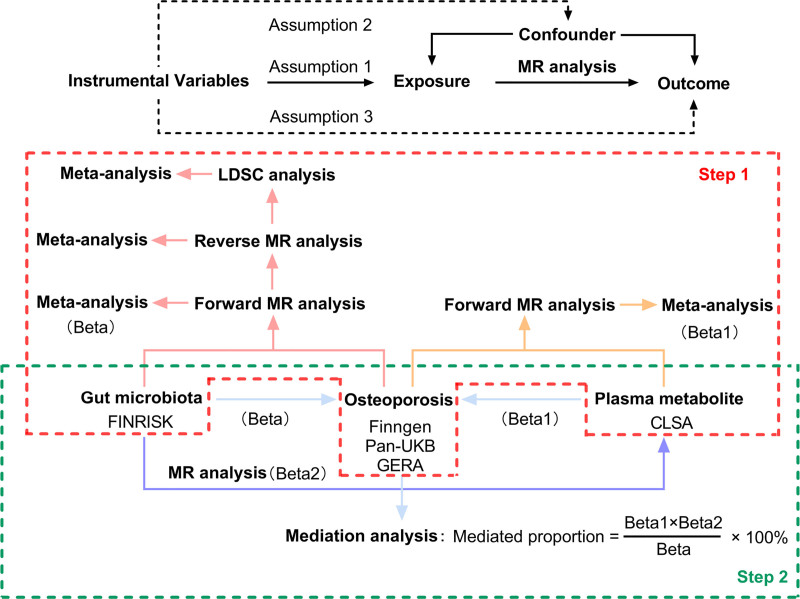
Three assumptions of MR analysis and overview of the study design. LDSC = linkage disequilibrium score regression, MR = Mendelian randomization. Adapted with permission from Zheng et al.^[9]^

All summary-level data analyzed in this study are publicly available. A schematic of the workflow is shown in Figure 1. In phase 1, we assessed bidirectional causal relationships between the gastrointestinal microbiome and osteoporosis via two-sample MR (forward and reverse) and quantified genome-wide genetic correlation using linkage disequilibrium score regression (LDSC). In parallel, we conducted forward MR to identify plasma metabolites associated with osteoporosis. Findings from MR and LDSC were then synthesized by meta-analysis to integrate evidence across datasets. In phase 2, we linked the prioritized microbiome taxa to their corresponding plasma metabolites and estimated mediation effects (ME) on osteoporosis using the product-of-coefficients approach.^[10]^ Our reporting followed the STROBE-MR recommendations.^[11]^ All contributing cohorts had prior ethics approval, and participants provided informed consent in accordance with the requirements of their local institutional review boards or ethics committees.

Summary statistics for gut microbiota were drawn from the FINRISK cohort, which included 5959 genotyped participants with metagenomic profiling, yielding 473 distinct taxa.^[12]^ Metabolite genome-wide association studies (GWAS) data – covering 1091 metabolites and 309 metabolite ratios – were obtained from the Canadian Longitudinal Study on Aging (CLSA; n = 8299, European ancestry).^[13]^ Osteoporosis summary data came from FinnGen GWAS Release 10, the Genetic Epidemiology Research on Aging (GERA) cohort, and Pan-UKB GWAS v0.4, totaling 21,353 cases and 853,313 controls of European ancestry.^[14–16]^ Summary-level data on gut microbiota were obtained from the FINRISK study, adjusted for age, sex, genotyping batch, and the top 10 genetic principal components. Similarly, plasma metabolites data from CLSA were adjusted for age, sex, hours since last meal or drink, genotyping batch, and the first 10 genetic principal components. Osteoporosis outcome data from FinnGen, Pan-UKB, and GERA were adjusted specifically for age, sex, genotyping batch or array, and principal components (10 for FinnGen, 8 for Pan-UKB, and 7 for GERA). These adjustments were performed by the original GWAS consortia prior to releasing summary-level data, effectively controlling for potential population stratification and confounding from demographic and technical variables. Detailed diagnostic definitions, covariates, and sample sizes are summarized in Table 1. We assessed MR power using an online calculator.^[17]^ Because the datasets originated from 5 independent resources, the potential for sample overlap was minimized.

**Table 1:** Detailed information on used summary-level data.

Exposure or outcome	Unit	Consortium	Participants included in analysis	Age (yr)	Male (%)	Adjustments	ICD/Phecode	PMID	Web source
Gut microbiota	SD	FINRISK	5959 European individuals	45.7 ± 11.5	50	Age, sex, genotyping batch and top 10 genetic principal components		35115689	https://www.ebi.ac.uk/gwas/
Plasma metabolite	SD	CLSA	8299 European individuals	63 (45–85)	52	Age, sex, hour since last meal or drink, genotyping batch and the first 10 genetic principal components		36635386	https://www.ebi.ac.uk/gwas/
Osteoporosis	One-unit in log-transformed odds ratio of Osteoporosis	FinnGen	8017 cases and 391,037 controls of European ancestry	53 ± 18	44	Sex, age, genotyping batch and 10 principal components	ICD-10: M80, M81, M82; ICD-9: 733.0	36653562	https://r10.finngen.fi/
		Pan-UKB	7937 cases and 411,038 controls of European ancestry	55.1 ± 7.6	48	Sex, age, genotyping array, and the first 8 principal components	Phecode: 743.1		https://pan.ukbb.broadinstitute.org/downloads/
		GERA	5399 cases and 51,238 controls of European ancestry	63 (19 to over 100)	42	7 derived principal components, sex, and age	ICD-9: 733.0	33893285	http://cg.bsc.es/gera_summary_stats/

SD = standard deviation , GERA = Genetic Epidemiology Research On Aging.

MR requires instruments that satisfy 3 core assumptions: genetic variants are strongly associated with the exposure; they are independent of factors that confound the exposure–outcome relationship; and they affect the outcome solely through the exposure.^[8]^ Using a genome-wide significance threshold of *P* < 5 × 10^−8^ and stringent linkage disequilibrium (LD) clumping (10,000 kb, *r*^2^ < 0.001), we initially identified instruments for 284 gut bacterial taxa (324 Single nucleotide polymorphisms (SNPs) in total) and 839 plasma metabolites (2230 SNPs in total). To reinforce instrument strength (assumption i), we additionally considered exposure SNPs at *P* < 5 × 10^−6^.^[18]^ For each candidate variant, we computed the *F*-statistic (*F* = β^2^/SE^2^) and removed SNPs with *F* < 10 to reduce weak-instrument bias.^[19,20]^ Effect alleles were harmonized across exposure and outcome datasets, excluding mismatched variants and ambiguous palindromic SNPs with Minor allele frequency (MAF) ≈ 0.5. We did not employ proxy variants for missing instruments because only a small proportion were unavailable and their omission had negligible impact. To assess horizontal pleiotropy, we applied MR pleiotropy residual sum and outlier (MR-PRESSO) and the MR-Egger intercept, and any MR estimates showing significant pleiotropy were excluded from the meta-analysis. To uphold the exclusion-restriction assumption (assumption iii), we also removed SNPs associated with the outcome at *P* < 5 × 10^−6^. After these filters, the forward MR stage encompassed 1178 plasma metabolites and 416 bacterial taxa. Comprehensive instrument lists for all taxa and metabolites are provided in Tables S1 and S2 (Supplemental Digital Content, https://links.lww.com/MD/P901).

### 2.2. Statistical analysis

Primary causal estimates were obtained with inverse-variance weighting (IVW) under a random-effects model. When all instruments satisfy the MR assumptions, IVW is the most statistically efficient approach and yields the most precise estimates.^[8]^ To assess robustness, we additionally applied 3 sensitivity methods: weighted median, MR-Egger, and MR-PRESSO. MR-Egger incorporates an intercept term to test for directional pleiotropy and typically provides more conservative estimates; a significant intercept indicates unbalanced pleiotropy.^[21]^ The weighted median estimator is less sensitive than IVW to a subset of invalid instruments and performs well when pleiotropy is modes.^[22]^ When pleiotropy is present or suspected, MR-PRESSO improves causal estimation by identifying and removing outlier variants that violate the exclusion restriction.^[23]^ Between-SNP heterogeneity was evaluated using Cochran *Q*, and horizontal pleiotropy was further examined via the MR-Egger intercept. Any instrument set with *P* < .05 for the MR-Egger intercept or the MR-PRESSO global test, as well as analyses with fewer than 4 SNPs (the minimum required by MR-PRESSO), was excluded from the meta-analysis. We then combined the IVW estimates with results from sensitivity methods using a fixed-effects meta-analytic model; IVW findings showing marked heterogeneity or inconsistency with sensitivity analyses were discarded. We next evaluated genome-wide genetic correlation between osteoporosis and the previously prioritized gut microbiome features using LDSC. Using HapMap3 as the LD reference, we removed non-SNP variants, SNPs with MAF < 0.01, duplicate records, and strand-ambiguous markers from the GWAS data. LDSC infers genetic correlation by relating LD scores to association statistics^[24]^; operationally, it regresses the cross-trait products of variant-wise z-scores on LD scores to estimate the correlation.^[25]^

We implemented a two-step MR framework for mediation analysis.^[26]^ To streamline inference, we first evaluated the relationships among bacteria, metabolites, and osteoporosis, and then examined associations between the prioritized gastrointestinal taxa and plasma metabolites. ME of microbiota on osteoporosis via metabolites were estimated using the product-of-coefficients approach,^[10]^ and the mediation proportion was calculated as ME divided by the total effect.^[27]^

To control multiple testing, Bonferroni correction was applied in the meta-analyses of MR results.^[28]^ For gut microbiota and plasma metabolites, respectively, causal associations were deemed significant when IVW *P*-values were <1.20 × 10^−4^ (0.05/416) and < 4.24 × 10^−5^ (0.05/1178), and considered suggestive when IVW *P*-values lay between these thresholds and 0.05. All analyses were performed in R (version 4.3.1) using the TwoSampleMR and meta packages.

## 3. Results

### 3.1. Gut microbiota and osteoporosis

**Figure 2.**
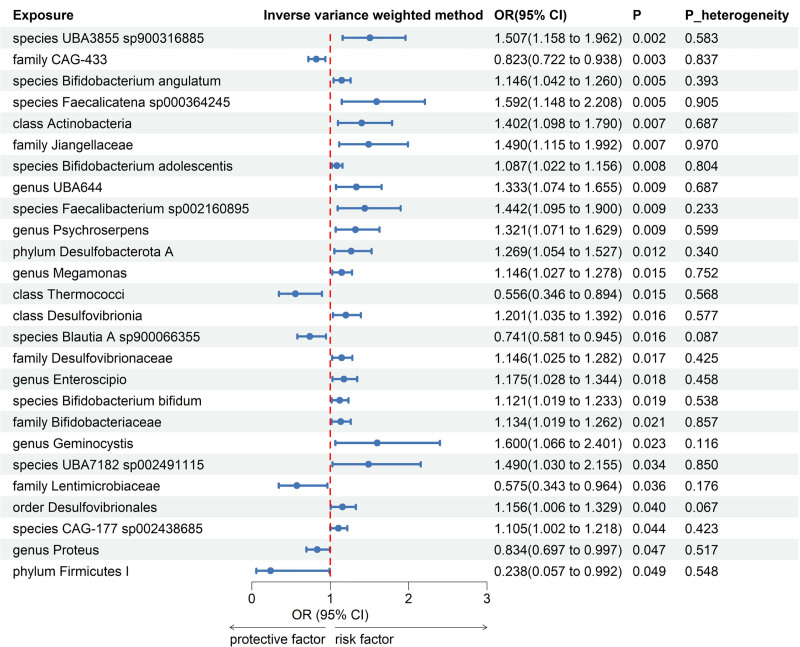
Forest plot of forward MR analysis between gut microbiota and osteoporosis. CI = confident interval, OR = odds ratio, P_heterogeneity = *P*-value of heterogeneity for meta-analysis.

**Figure 3.**
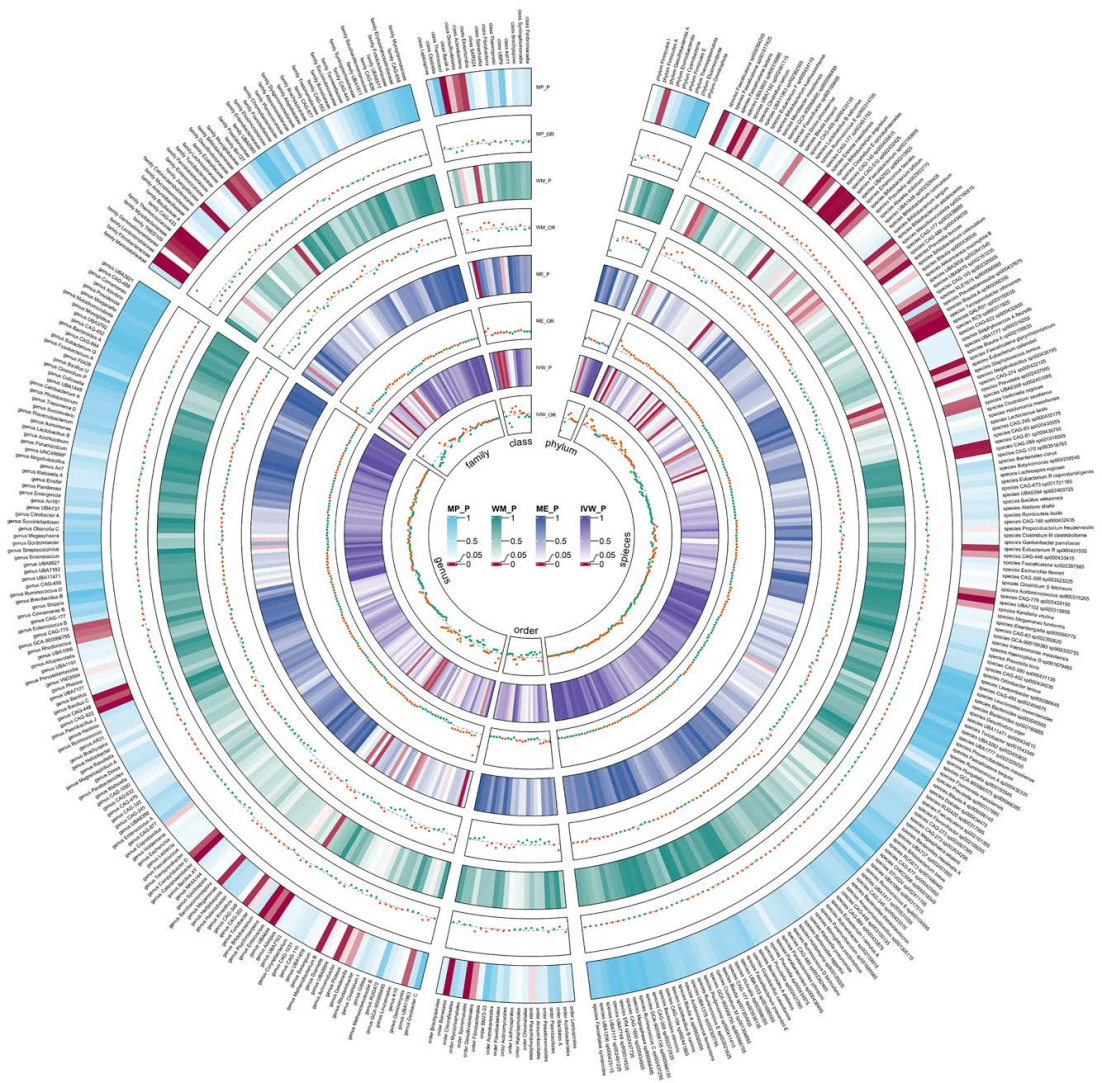
Circular heat map of meta-analysis of genetic correlation between gut microbiota and osteoporosis. The color variations represented the size of the *P*-value. The scatter plots reflect OR, with OR > 1 labeled red and OR < 1 labeled green. IVW = inverse-variance weighted, ME = MR-Egger, MP = MR-PRESSO, OR = odds ratio, WM = weighted median.

Meta-analyses of 416 bacterial taxa were conducted (Table S3, Supplemental Digital Content, https://links.lww.com/MD/P901). Finally, we identified 26 gut microbiomes suggestively linked to osteoporosis. The combined IVW estimates showed that genetically predicted phylum *Desulfobacterota A* (OR = 1.269; 95% CI = 1.054, 1.527; *P* = .012), order *Desulfovibrionales* (OR = 1.156; 95% CI = 1.006, 1.329; *P* = .040), family *Desulfovibrionaceae* (OR = 1.146; 95% CI = 1.025, 1.282; *P* = .017), and 17 other gut microbiomes were potentially linked to an increased risk of osteoporosis. Furthermore, genetically predicted genus *Proteus* (OR = 0.834; 95% CI = 0.697, 0.997; *P* = .047) and 5 other gut microbiomes were potentially linked to a decreased risk of osteoporosis (Fig. 2). All main results and sensitivity analyses were depicted in Figure 3.

**Figure 4.**
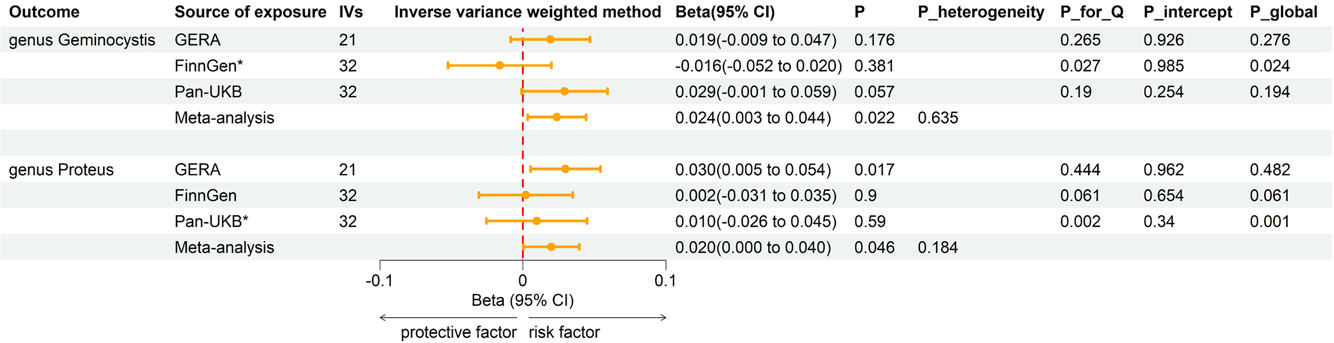
Forest plot of reverse MR analysis between identified gut microbiota and osteoporosis. CI = confident interval, IVs = instrumental variables, P_heterogeneity, *P*-value of heterogeneity for meta-analysis, P_Q = *P*-value for Cochran *Q* test, P_intercept = *P*-value for MR-Egger intercept test, P_global = *P*-value for Global test, SNPs = single nucleotide polymorphisms. * Excluded from the meta-analysis due to SNPs < 4 or significant pleiotropy.

Reverse MR analyses were conducted between osteoporosis and previously identified 26 gut microbiomes (Table S4, Supplemental Digital Content, https://links.lww.com/MD/P901). The combined IVW estimates revealed that genetically predicted osteoporosis was potentially linked to an increased abundance of genera *Geminocystis* (Beta = 0.024; 95% CI = 0.003, 0.044; *P* = .022) and *Proteus* (Beta = 0.0199; 95% CI = 0.0004, 0.0395; *P* = .046) (Fig. 4).

Given the modest heritability and limited sample sizes, only 22 of the previously prioritized gut microbial traits qualified for LDSC analysis. The aggregated LDSC estimates indicated a possible positive genetic correlation between genetically proxied *Bifidobacterium angulatum* and osteoporosis (Rg = 0.202; 95% CI = 0.013–0.390; *P*_Rg = .036). Complete results for all genetic associations are provided in Table S5 (Supplemental Digital Content, https://links.lww.com/MD/P901).

### 3.2. Plasma metabolites and osteoporosis

**Figure 5.**
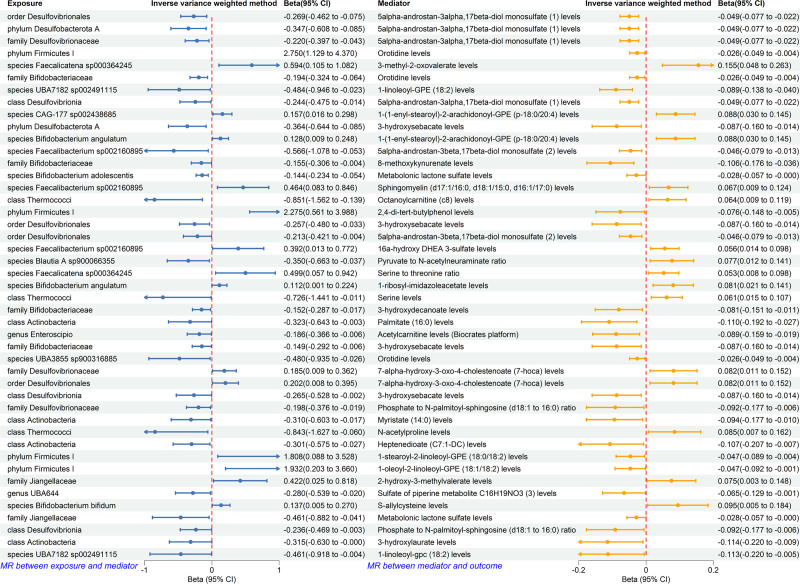
Forest plot of MR analysis between identified gut microbiota and identified plasma metabolites, and between identified plasma metabolites and osteoporosis. CI = confident interval..

In the forward MR screen, we identified 91 plasma metabolites with genetically predicted effects on osteoporosis (Table S6, Supplemental Digital Content, https://links.lww.com/MD/P901). We then performed 2366 MR tests to evaluate links between the prioritized bacterial taxa and these metabolites (26 taxa × 91 metabolites; Table S7, Supplemental Digital Content, https://links.lww.com/MD/P901). In total, 45 microbiome–metabolite pairs qualified for mediation analysis (Fig. 5). Among these, genetic predisposition to phylum *Desulfobacterota A* (Beta = −0.347; 95% CI = −0.608, −0.085; *P* = .009), order *Desulfovibrionales* (Beta = −0.269; 95% CI = −0.462, −0.075; *P* = .007), and family *Desulfovibrionaceae* (Beta = −0.220; 95% CI = −0.397, −0.043; *P* = .015) was causally linked to 5alpha-androstan-3alpha,17beta-diol monosulfate (3α-diol MS) levels.

### 3.3. Mediation analysis

Mediation analyses were conducted for 45 specific pairings of gut microbiomes and plasma metabolites (Table S8, Supplemental Digital Content, https://links.lww.com/MD/P901). Specifically, the phylum *Desulfobacterota A* indirectly influenced osteoporosis through 3α-diol MS levels, with an ME of 0.017 (95% CI = 0.001, 0.033, *P* = .037), the order *Desulfovibrionales* indirectly influenced osteoporosis through 3α-diol MS levels, with an ME of 0.013 (95% CI = 0.001, 0.025, *P* = .032), and the family *Desulfovibrionaceae* indirectly influenced osteoporosis through 3α-diol MS levels, with an ME of 0.0109 (95% CI = 0.0002, 0.0215, *P* = .046). The mediated proportions were 7.18%, 9.15%, and 7.99% respectively (Table 2).

**Table 2:** Mediation analysis between gut microbiota, plasma metabolites, and osteoporosis.

Gut microbiota	Metabolite	Total effect EO (95% CI)	Effect EM (95% CI)	Effect MO (95% CI)	Mediation effect (95% CI)	Mediated proportion
Order *Desulfovibrionales*	3α-diol MS levels	0.1453 (0.0064–0.2842)	−0.2687 (−0.4624 to −0.0751)	−0.0494 (−0.0772 to −0.0216)	0.0133 (0.0011–0.0254) *P* = .032	9.15%
Phylum *Desulfobacterota A*	3α-diol MS levels	0.2381 (0.0529–0.4233)	−0.3466 (−0.6078 to −0.0854)	−0.0494 (−0.0772 to −0.0216)	0.0171 (0.001–0.0332) *P* = .0372	7.18%
Family *Desulfovibrionaceae*	3α-diol MS levels	0.1364 (0.0247–0.2481)	−0.2198 (−0.3966 to −0.043)	−0.0494 (−0.0772 to −0.0216)	0.0109 (0.0002–0.0215) *P* = .0458	7.99%

3α-diol MS = 5alpha-androstan-3alpha,17beta-diol monosulfate, EM = exposure to mediator, EO = exposure to outcome, MO = mediator to outcome.

### 3.4. Sensitivity analysis, pleiotropy, and heterogeneity

During data processing, we excluded IVW estimates that were inconsistent with the sensitivity analysis (weighted median, MR-Egger, and MR-PRESSO) or exhibited significant pleiotropy (*P* for intercept < .05 or *P* for global test < .05) to maintain the stability and validity of our findings. Most SNPs (Cochran *Q* test) and the meta-analysis results showed no or mild heterogeneity, demonstrating the robustness of our analyses.

## 4. Discussion

This study investigated the relationship between gut microbiota, plasma metabolites, and osteoporosis using GWAS and MR. The research utilized data from Pan-UKB, FinnGen, and GERA, encompassing over 21,000 cases and 853,000 controls. Findings revealed 26 gut microbiomes linked to osteoporosis, with specific taxa like *Desulfobacterota A* associated with increased risk and *Proteus* with decreased risk. Additionally, plasma metabolites like 3α-diol MS were causally associated with osteoporosis, and mediation analysis indicated that gut microbiota influenced osteoporosis through these metabolites, mediating up to 9.15% of the effect. These results highlight the complex interaction between gut microbiota and bone health, suggesting potential therapeutic targets.

More than 90% of the makeup of the gut microbiota community is accounted for by the phyla *Firmicutes*, *Bacteroidetes*, *Proteobacteria*, and *Actinobacteria*.^[29]^ The multifaceted effects of gut microbiota have indeed shown compelling evidence linking its dysregulation, known as dysbiosis, to a spectrum of pathologies, including dyslipidemia, insulin resistance, systemic inflammation, and abnormal bone metabolism.^[30–33]^ For instance, dysbiosis can impair intestinal barrier function, leading to increased bone resorption and decreased bone formation, contributing to osteoporosis. In a study, fecal microbiota transplantation from osteoporotic rats to healthy rats induced significant bone loss and impaired intestinal structure.^[34]^ Additionally, dysbiosis aggravates inflammation, a key factor in bone turnover. Studies show that the gut microbiome’s modulation of the immune system can influence bone health, with specific microbial changes linked to osteoporosis.^[35]^ Metabolomics analyses indicate that gut microbiota dysbiosis affects amino acid metabolism, which is crucial for bone health. Specific bacteria and their metabolic pathways have been associated with osteoporosis in large cohort studies.^[36]^

Observational studies indicate that individuals with osteoporosis exhibit altered gut microbiota compositions, particularly an increased *Firmicutes*/*Bacteroidetes* ratio, which is linked to bone health deterioration. For instance, the abundance of *Bacteroides* is often elevated, while beneficial bacteria such as *Lactobacillus* and *Veillonella* are reduced in osteoporotic patients.^[37]^ Animal studies further support these findings, showing that gut dysbiosis can exacerbate bone loss. For example, ovariectomized mice, a model for postmenopausal osteoporosis, exhibit a notable increase in the *Firmicutes*/*Bacteroidetes* ratio, decreased bone formation, and increased bone resorption.^[35]^ These studies highlight the role of gut microbiota in modulating bone metabolism through immune system interactions and nutrient absorption. MR studies have provided causal evidence linking specific gut microbiota to bone mineral density (BMD). Higher levels of the genus *Prevotella9* and family *Prevotellaceae* are causally associated with increased lumbar spine and forearm BMD, respectively. Conversely, certain genera like *Blautia* and *Parabacteroides* are associated with lower BMD and higher osteoporosis risk.^[38]^ Moreover, dysbiosis in the gut microbiota, characterized by increased diversity and altered functional pathways, is linked to osteoporosis, with specific taxa such as *Actinobacillus*, *Blautia*, and *Oscillospira* positively associated with the disease, while *Veillonellaceae* and *Collinsella* show inverse relationships.^[36]^ In conclusion, evidence from various research methodologies indicates that specific gut microbiomes play a significant role in osteoporosis. These findings suggest potential therapeutic targets within the gut microbiome for preventing and treating osteoporosis through dietary modifications, probiotics, and prebiotics to restore healthy gut microbiota balance.^[39]^

Family *Desulfovibrionaceae*, belonging to the phylum *Desulfobacterota A*, specifically within order *Desulfovibrionales*, are known for their sulfate-reducing capabilities, which means they can use sulfate as an electron acceptor during respiration.^[40]^ Species whin family *Desulfovibrionaceae* like *Desulfovibrio desulfuricans*, *Desulfovibrio vulgaris*, and *Desulfovibrio aminophilus* are all capable of reducing sulfate through reducing nitrate, recovering electrons, or degrading amino acids.^[41–43]^ This was in accordance with our findings, which demonstrated that family *Desulfovibrionaceae* was related with lower 3α-diol MS levels. 3α-diol MS is a sulfate-conjugated form of 3alpha-diol, a key metabolite derived from dihydrotestosterone (DHT).^[44]^ Androgens, including testosterone and its metabolites like DHT and 3α-diol, have been shown to influence bone density positively. These hormones stimulate bone formation and inhibit bone resorption, processes critical for maintaining bone mass.^[45]^ Androgens exert their effects on bone through androgen receptors present in bone cells. The activation of these receptors by metabolites like 3α-diol is crucial for maintaining bone health. Lower levels of these metabolites can lead to reduced receptor activation, impairing bone maintenance and leading to osteoporosis.^[46,47]^ Additionally, studies have shown that levels of 3α-diol decline with age, which corresponds with the observed increase in osteoporosis incidence among the elderly. This decline reduces the anabolic effects on bone, leading to decreased bone density and higher fracture risk.^[48]^

This study has several notable strengths. First, we employ MR to support causal inference, thereby reducing confounding and reverse causation. Second, we draw on large, publicly available GWAS resources – encompassing more than 21,000 osteoporosis cases and over 853,000 controls – which affords ample statistical power and reliability. Third, we interrogate the microbiota–osteoporosis relationship in both directions (forward and reverse MR) and evaluate mediation through plasma metabolites, offering a more granular view of microbiota–bone pathways. Finally, meta-analytic synthesis, a suite of sensitivity analyses (MR-Egger, weighted median, MR-PRESSO), and LDSC-based genetic correlation testing strengthen robustness by detecting and addressing pleiotropy and heterogeneity, supporting the stability and validity of our results.

Evaluating our findings requires an awareness of several limitations. Although our study employed rigorous MR analysis methods and utilized large-scale datasets to control for potential biases, it remains susceptible to residual confounding due to population stratification. Specifically, genetic background, environmental factors, lifestyle habits, diet, medication use, and other unmeasured confounding variables were not fully accounted for in the publicly available summary-level data utilized. Furthermore, demographic details such as age, gender, and other subgroup characteristics were limited, preventing detailed stratification analyses. This limitation could impact the generalizability of our results, as different demographic groups may exhibit varied associations. Future studies should prioritize collecting detailed demographic data and include individuals from diverse ethnic backgrounds to explore whether associations between gut microbiota, plasma metabolites, and osteoporosis differ across various populations and demographic categories, including race, age, and gender. Such studies could enhance the generalizability of findings and inform tailored prevention and intervention strategies. Another potential limitation pertains to the comprehensiveness of the databases utilized. Although we included publicly accessible GWAS data encompassing a large number of participants and a wide array of genetic variants, we cannot fully exclude the possibility that some relevant genetic variants or microbial taxa influencing osteoporosis were not captured due to limitations inherent in current genomic and metagenomic databases. Future studies should aim to incorporate more comprehensive and updated genetic and microbiome datasets, potentially integrating metagenomic sequencing data and larger cohorts, to ensure better representation of the diversity and complexity of gut microbiota and related genetic variants. Finally, MR is typically optimal for assessing genetic exposures fixed from birth, ensuring that genetic variants precede and predict the outcome. However, the gut microbiome is dynamic and subject to substantial changes influenced by dietary patterns, medication use, lifestyle, and environmental factors throughout life. Therefore, genetic variants associated with gut microbiome composition might not fully represent lifetime microbial exposures or functional shifts in the microbiome over time. This temporal variability can introduce additional complexity and potential bias into the interpretation of MR results linking gut microbiota to health outcomes, including osteoporosis. To further validate and extend the hypotheses generated from this MR analysis, future research should include prospective longitudinal cohort studies involving repeated sampling of gut microbiota, plasma metabolites, and detailed dietary and lifestyle information over time to confirm temporal causality. Additionally, functional experimental studies using animal models or clinical trials that specifically manipulate identified microbiota taxa or plasma metabolites (e.g., Desulfobacterota, Desulfovibrionaceae, or 3α-diol MS) would provide mechanistic insights into the observed associations. Further validation in multi-ethnic and diverse demographic cohorts would be valuable for confirming the generalizability of these findings. Lastly, integrating multi-omics approaches, such as transcriptomics, proteomics, and advanced metabolomics, can provide comprehensive insights into the biological mechanisms linking gut microbiota with bone health, facilitating the development of targeted microbiome-based diagnostics and therapeutic interventions.

## 5. Conclusion

This study underscores the significant impact of gut microbiota on osteoporosis, identifying 26 gut microbiomes associated with the disease, with taxa such as *Desulfobacterota A* linked to increased risk and Proteus to decreased risk. Moreover, plasma metabolites, notably 3α-diol MS, were found to mediate the relationship between gut microbiota and osteoporosis, explaining up to 9.15% of the effect. These findings highlight the intricate interplay between gut microbiota, plasma metabolites, and bone health, providing valuable insights into potential therapeutic targets. Future research should aim to elucidate the precise mechanisms underlying these interactions. Additionally, exploring therapeutic interventions that modify the gut microbiota could help prevent and treat osteoporosis.

## Acknowledgments

The authors express their gratitude toward all the participants and investigators who contributed to the GWASs involved in the present study by generously sharing the summary-level data.

## Author contributions

**Conceptualization:** WeiDong Zhu.

**Data curation:** WeiDong Zhu.

**Formal analysis:** WeiDong Zhu.

**Investigation:** ShuWei Ma.

**Methodology:** ShuWei Ma.

**Project administration:** JinNan Yin.

**Resources:** XuWen Zheng.

**Software:** XuWen Zheng.

**Supervision:** XuWen Zheng, JinNan Yin.

**Writing – original draft:** WeiDong Zhu.

**Writing – review & editing:** ShuWei Ma, XuWen Zheng, JinNan Yin.

## Supplementary Material


